# Does Ultraviolet Radiation Exhibit Antimicrobial Effect against Oral Pathogens Attached on Various Dental Implant Surfaces? A Systematic Review

**DOI:** 10.3390/dj10060093

**Published:** 2022-05-31

**Authors:** Fahad A. Abdullatif, Mansour Al-Askar

**Affiliations:** Department of Periodontics and Community Dentistry, College of Dentistry, King Saud University, Riyadh 11545, Saudi Arabia; fahad.dmd@gmail.com

**Keywords:** ultraviolet, dental implant, oral pathogen, peri-implantitis

## Abstract

**Background**: Dental implant therapy is currently identified as the most effective treatment for edentulous patient. However, peri-implant inflammations were found to be one of the most common complications that leads to the loss and failure of dental implantation. Ultraviolet (UV) radiation has been proposed to enhance bone integration and reduce bacterial attachment. In this study, we aimed to systematically review the current evidence regarding the antimicrobial effect of UV on different dental implant surfaces. **Methods:** Five databases including PubMed, Scopus, Web of science, VHL, and Cochran Library were searched to retrieve relevant articles. All original reports that examined the effect of the application of UV radiation on dental implants were included in our study. **Results:** A total of 16 in vitro studies were included in this systematic review. Polymethyl methacrylate UV radiation has induced a significant decrease in bacterial survival in PMMA materials, with an increased effect by modification with 2.5% and 5% TiO_2_ nanotubes. UV-C showed a superior effect to UV-A in reducing bacterial attachment and accumulation. UV wavelength of 265 and 285 nm showed powerful bactericidal effects. UV of 365 nm for 24 h had the highest inhibition of bacterial growth in ZnO coated magnesium alloys. In UV-irradiated commercially pure titanium surfaces treated with plasma electrolytic oxidation, silver ion application, heat or alkali had shown significant higher bactericidal effect vs non-irradiated treated surfaces than the treatment with any of them alone. UVC and gamma-ray irradiation increased the hydrophilicity of zirconia surface, compared to the dry heat. **Conclusion:** UV radiation on Ti surfaces exhibited significant antibacterial effects demonstrated through the reduction in bacterial attachment and biofilm formation with suppression of bacterial cells growth. Combination of UV and treated surfaces with alkali, plasma electrolytic oxidation, silver ion application or heat enhance the overall photocatalytic antimicrobial effect.

## 1. Introduction

Dental implant is a routine treatment for edentulous patients with an increase in the prevalence by 14% per year in the United States [1]. Like the natural predecessor, the dental implant may become prone to plaque-associated inflammatory conditions. Peri-implant mucositis and peri-implantitis are the most common complications of dental implants estimated at 29.48% and 9.25% of dental implants, respectively [2]. The etiology of both conditions is bacterial accumulation on the implant and restoration surfaces. Peri-implant mucositis is the reversible form where only the soft tissue exhibits bleeding on probing, erythema, swelling and/or suppuration while peri-implantitis is defined as a state of inflammation that occurs in the osseointegrated complex where activation of host response and an increase of osteoclastic activity occurs mainly due to pathogenic bacteria. It is well known that periodontal pathogenic bacteria play a role in the development of peri-implantitis through various kinds of implant surfaces [3]. Furthermore, a recent meta-analysis that evaluated 28 studies that used PCR-based methods to detect the microbiological content of peri-implantitis sites in comparison with healthy implants revealed that *Aggregatibacter actinomycetemcomitans* and *Prevotella intermedia* were associated with disease with a log odds-ratio of 4.04 and 2.28, respectively. In addition, *Porphyromonas gingivalis*, *Tannerella forsythia* and *Treponema denticola* were also found to be associated with the disease [4]. It is currently unclear whether these bacteria cause peri-implant disease or they emerge as a result of the disease. Rakic et al. conducted a systematic review on the microbiologic profile of peri-implantitis in humans and summarized key findings in that peri-implantitis is a complex infection with variable organisms. However, the profile of this inflammatory condition consists mainly of Gram-negative anaerobic periodontal pathogens [5].

Since Gram-negative anaerobic bacteria are associated with peri-implantitis, decontamination of implant surface is the main preventive measure in the management of peri-implantitis. Several treatment options have been suggested, such as mechanical debridement, chemical decontamination and antimicrobial drugs. However, curettes and ultrasonic devices have failed to obliterate bacterial counts in peri-implantitis [6]. Furthermore, chemical decontamination with hydrogen peroxide and chlorhexidine gluconate was found to be inferior to mechanical debridement [7]. The efficacy of antibiotic management is still debatable and depends on implant surface characteristics [8,9]. Therefore, laser and photodynamic therapy were also used in the decontamination of dental implants to avoid alterations in the implant surface morphology [10]. In spite of the clinical improvement in peri-implant mucositis, the recent evidence remains inconclusive due to the lack of standardized methodology and parameters [11]. 

Ultraviolet radiation (UV) is a non-visible, high-frequency, short-wavelength light that naturally emanates from the sun or synthetic sources, that is also bactericidal. UV radiation can be further divided according to wavelength into: UV type A (315–400 nm), UV type B (280–315 nm) and UV type C (100–280 nm) [12]. In addition, a 7-year prospective study on healthy humans receiving UV-treated implants revealed a 100% success rate even in areas where the alveolar bone was atrophic and guided bone regeneration was performed simultaneously with the implant surgery [13]. UV-treated implants had statistically significant less bone loss compared to non-UV-treated implants in a dog model with experimental peri-implantitis [14]. Furthermore, UV-treated titanium surfaces exhibited an antimicrobial effect through enhanced photocatalytic properties that positively decreased periodontal pathogenic bacteria [15]. On the other hand, ultraviolet irradiation (UV) photofunctionalization has been defined as the physicochemical and biological modification that occurs on treated titanium surfaces, which enhances titanium surfaces to hydrophilic, superhydrophilic surfaces and improves osseointegration in commercially pure titanium surfaces (CPT) [16,17]. Of note, CPT is mainly used in dental implants with higher survival rates reaching 99%. UV radiation is well known to have an antimicrobial effect through photochemical reactions that affect bacteria DNA which inhibits proliferation and survival. Recently, UVC with a wavelength of 260 nm and Light Emitting Diode (LED)-based UV showed an ability to remove contaminant hydrocarbons on different titanium dental implant surfaces [18,19]. In the current study, we aimed to systematically review the recent evidence regarding the antimicrobial effect of UV exposure on different dental implant surfaces.

## 2. Materials and Methods

This current systematic review of the literature was performed following the Preferred Reporting Items for Systematic Reviews and Meta-Analysis (PRISMA) recommendations. The study protocol was approved by PROSPERO (298587).

### 2.1. PICO Question

(1) Population: dental implant surfaces that are attached to pathogenic bacteria. (2) Intervention: ultraviolet radiation. (3) Comparator: UV-treated surfaces vs. non-UV-treated surfaces. (4) Outcome: antimicrobial efficacy.

### 2.2. Study Design:

The literature was collected in July 2021. Published studies were identified by searching the electronic databases Medline, Embase, Web of Science and the Cochrane Library. The search strategy was built using a combination of keywords and MeSH terms for the two main axes of the research question: (1) UV technologies; and (2) dental implant. We built our search strategy with terms related to UV light (“UV”, “UVC”, “UVA”, “UVB”, “ultra violet”, “ultra-violet”, “ultraviolet rays”,“photofunctionalization”) and terms related to dental implant (“dental implant”, “peri-implantitis”, “periimplantitis”, “Peri-implant mucositis”, “peri-mucositis”, “implant”, “titanium disks”). The present systematic review and the search strategy were established with no language and publication time restrictions. In addition, manual screening of the reference lists of all included studies was performed. Expert authors were contacted for information on ongoing studies and unpublished data. 

All original studies, case reports, case series studies, in vitro and in vivo studies that investigated the effect of application of ultraviolet irradiation combined or not combined with other materials on dental implants were included. Poster publications, commentaries, letters, review articles, thesis, conference, and book chapter’s full-text not available or not in English were excluded.

Two reviewers independently collected the data from full texts that met our criteria. Pilot-tested data extraction sheet was first developed before the final version. Data extracted included study characteristics, sample size, implant type, follow-up duration, success rates, and bactericidal effect on different bacteria pathogens. 

## 3. Results

**Figure 1:**
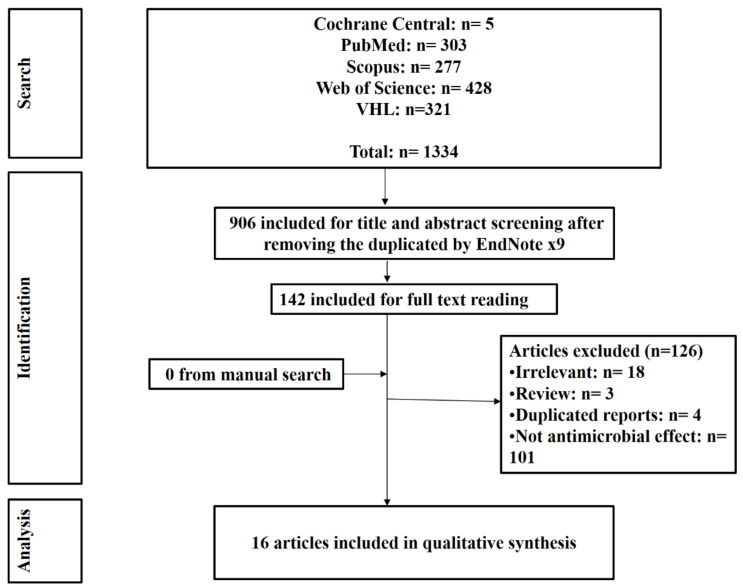
Flow diagram of included studies.

Our search revealed 1334 papers, 906 underwent title and abstract screening, 142 were eligible for full-text scanning. Finally, we included 16 studies (Figure 1) in this systematic review and the detailed data of included studies are listed in Table 1. All included studies are in vitro investigations of the antimicrobial effect of UV rays on various dental implant materials. The investigated implant materials included polymethyl methacrylate (PMMA) with or without titanium dioxide nanotubes modification, commercially pure titanium (CPT) discs, titanium dioxide (TiO_2_), zirconia, novel nanopeptide (NP) adhesive, and magnesium alloy with the single zinc oxide (ZnO). The studies included in the current review illustrated wide variation in terms of the used UV light wavelength and the time of exposure. The measurement used for the antimicrobial effect is mainly the colony-forming unit (CFU) along with the Biofilm assay and Biofilm Organization by scanning electron microscopy (SEM). 

### 3.1. Polymethyl Methacrylate (PMMA)

For UV radiation on PMMA specimens, a single study compared the conventional PMMA with modified hydrothermally synthesized titanium dioxide nanotubes. UV irradiation has induced a significant decrease in survival of *candida Albicans*, *Lactobacillus acidophilus*, *and Streptococcus mutans* more than in non-irradiated surfaces which increased with the increased ultraviolet light energy exposure. In addition, modifications with 2.5% and 5% TiO_2_ nanotubes were reported to improve the antimicrobial properties [20]. In agreement, Binns et al. found a significant decrease in *candida Albicans* survival with the increased exposure to ultraviolet radiation. Of note, the antimicrobial effect of UV exposure for 5 min was found to be like the effect of 3.8% sodium perborate on PMMA surfaces [21].

### 3.2. Commercially Pure Titanium (CPT)

In CPT, there is a significant reduction in attached bacterial cells to UV-irradiated titanium surfaces compared to non-irradiated surfaces. Furthermore, biofilm formation was examined under confocal laser scanning microscopy which revealed biofilm thickness of 16 μm and 8 μm for non-irradiated surfaces and irradiated surfaces, respectively [22]. Upon differentiating the antimicrobial efficacy between UV-A and UV-C with an intensity of 500 J/cm^2^, UV-C showed a superior effect to UV-A in reducing bacterial attachment and accumulation [23]. Moreover, irradiated anatase TiO_2_ powder that could be placed in a peri-implant pocket, showed a continuous photocatalytic and antimicrobial effect against *Porphyromonas gingivalis* [24]. Johnson et al. examined the bacterial attachment on CPT with different ultraviolet intensities and revealed an insignificant change in bacterial attachment with irradiation by 1 μW/cm^2^ and 8 μW/cm^2^ UV-A. However, high UV-A intensities with 23 μW/cm^2^ showed a roughly complete reduction in *Staphylococcus aureus* attachment for both anodized and un-anodized CPT [25]. 

In terms of treated titanium materials, a significant decrease in the CFU counts was also reported in irradiated CTP surfaces treated with plasma electrolytic oxidation (PEO) vs non-irradiated treated PEO CTP surfaces [26]. Additionally, treatment with silver ion application combined with UV-A light exposure had a significantly higher antimicrobial effect than the treatment with any of them alone [27]. Furthermore, UV combined with heat showed inhibition of bacterial attachment and biofilm formation with antibacterial rates of 60% and 96% after 1 and 6 h, respectively, on crystallized nanostructured titanium [28]. Likewise, UV-irradiated alkali-treated titanium with nanonetwork structures (TNS) also showed a similar reduction up to 6 h [29]. Lee et al. compared irradiated and non-irradiated titanium machined surfaces, heat-treated and anodized surfaces, combined with whole saliva or phosphate-buffered saline for 2 h, irradiated heat-treated and anodized surfaces had greater antibacterial effects than irradiated titanium machined surfaces. However, the presence of saliva coating increased bacterial survival rates in all experimental titanium surfaces that may affect the photocatalytic effect of UV [30]. 

### 3.3. Other Materials

Regarding zirconia dentures sterilization methods, the exposure to UVC and gamma-ray irradiation increased the hydrophilicity of the zirconia surface, while the dry heat samples showed the lowest amount of bacteria growth [31]. On the novel nanopeptide (NP) adhesive surfaces, irradiation with a UV dose of 8.4 J/cm^2^ had a great reduction in the number of biofilm bacteria, and UV dose of 43 J/cm^2^ had a five-fold greater effect; however, UV-A dose of 16 J/cm^2^ was insufficient to affect the viability of the biofilm [32]. 

Exposing ZnO-coated magnesium alloys for UV of 365 nm from the mercury lamp for 24 h had the highest inhibition of bacterial growth of cells compared to naked magnesium alloys and non-irradiated ZnO-coated magnesium alloys [33]. In an attempt to study the effect of different UV wavelengths in vitro against periodontal and peri-implant pathogenic bacteria (*P. gingivalis*, *Prevotella intermedia*, *Fusobacterium nucleatum*, *A. actinomycetemcomitans*), UV wavelengths of 265 and 285 nm showed the highest bactericidal effect. On the other hand, partial growth suppression of bacterial cells was noted when a UV wavelength of 310 nm was administered. On the contrary, colony-forming units results were insignificant between UV wavelengths of 365 nm, 448 nm and control [34].

## 4. Discussion

The current study comprehensively reviewed the recent body of evidence on the antimicrobial features of UV rays by comparing the results of previous studies. the emerging theme is that UV light has a significant antimicrobial effect, which depends on many factors, including the UV dose and duration of exposure and material of the implant being treated or not. 

Among the epidemiological studies and increased rates of dental implants, a clear definition and classification of periodontal and peri-implant diseases were developed in 2017 by world workshops, focusing on visual signs of inflammation, bleeding on probing, with/without suppuration, increased probing depth more than 6 mm, increased radiographic bone loss more than 3 mm in the apical part of the coronal intraosseous portion of the implant [35]. However, in a recent critical review, the authors suggested that the prevalence of peri-implantitis may be overestimated because of wide thresholds for bone loss and a lack of standardized case definitions [36]. Several causative organisms have been identified to be involved in peri-implantitis such as *Porphyromonas gingivalis*, *Prevotella intermedia/nigrescens*, *Actinobacillus actinomycetemcomitans*, *Staphylococcus*, *and Candida albicans* [37]. Several risk factors may be associated with peri-implantitis including smoking and diabetes, where smoking had increased the risk of peri-implantitis by two times, meanwhile, hyperglycemia increased the risk by three times [38,39]. The role of oral hygiene is of significant importance and is linked with lower plaque and bleeding indexes on dental implants. It is thought that inadequate oral hygiene maintenance acts as a secondary reason for implant failure [40].

Regarding the effect of UV on the growth and gene expression of different bacteria species, Aung et al. found that ultraviolet light-emitting diodes (LED-UV) could produce nonthermal devitalization of bacteria on CPT denture surfaces, and may not produce thermal damage to the surrounding periodontal tissues which could be considered as one of the advantages of using these UV diodes for bactericidal purposes [34]. This comes in contrast to the thermal devitalization caused by using relatively high power lasers, such as CO2, Nd: YAG, Diode, and Er: YAG lasers, resulting in thermal damage to host tissues and cells. Furthermore, the UVC and UVB light with shorter wavelengths such as 265, 285, and 310 nm had the strongest bactericidal effect [41]. This could be explained by the devastating effects of shorter wavelength UV irradiation on DNA pyrimidine dimer lesions that in turn inhibit cell proliferation and induce apoptosis, eventually leading to cell death [42].

Several studies demonstrated that the antimicrobial properties of UV on titanium are referred to as the photocatalytic response of TiO_2_. In a photocatalysis system under a UV source, the electron of the photocatalyst gets stimulated and the extra energy of this excited electron creates electron (e-)-hole (h+) pairs. The resulted electrons and holes react with water and oxygen and produce reactive oxygen species (ROS), such as (O2•−), (•OH), and H_2_O_2_, which can decompose nearby organic compounds [43]. Moreover, the higher concentration of TiO_2_ nanotubes leads to higher ROS and consequently greater antibacterial properties [21].

In one included study, Pantaroto et al. assessed the UV effect on TiO_2_ treated by radiofrequency magnetron sputtering to obtain anatase (A-TiO_2_), rutile (R-TiO_2_), or a mixture (anatase + rutile) (M-TiO_2_), and a significant antibacterial effect was noted in both A-TiO_2_ and M-TiO_2_ films on multispecies biofilm after 1 h of irradiation. On the other hand, no antibacterial effect was noted in R-TiO_2_ films [44]. The purpose of these three crystalline TiO_2_ films is to create interspecies bacterial interactions and spatially organized biofilms that resemble the oral environment. 

Further, combination treatment with UV irradiation may result in more effective outcomes. Applying UV with hydrogen peroxide or caffeic acid was found to increase the efficacy of antimicrobials actively and enhance bioactivity and osseointegration of titanium implant surfaces [45]. Another study anodized the titanium surfaces with mixed-acid electrolyte solutions including sulfuric acid, phosphoric acid, and hydrogen peroxide, however, no difference was noted between anodized and unionized CPT with high UVA intensities. Moreover, significantly greater human blood plasma proteins and albumin adsorption were observed in irradiated CPT treated with PEO compared with non-irradiated PEO-treated CTP [26]. Since silver ions are well known to have an antimicrobial effect through interaction with the ribosome and alternating ATP production [46], Tenkumo et al. found the combination between UV and silver ions may increase hydroxyl radicals that enhance the bactericidal effect [27]. In addition, chlorohexidine mouthwash was found to have a profound antiseptic effect against the bleeding index and plaque index of human subjects [47]. Such results may provide a synergistic effect when combined with UV treatment.

UV treatment application may not be limited on the implant surface only. It is well known that implant abutment connections may harvest pathogenic bacteria that could lead to peri-implant infection [48]. UV treatment on implant abutments could exhort less bacterial contamination and, hence, enhance the implant survival rate. Furthermore, UV application may be recommended in cases with atrophic jaws that require reconstruction using bone grafts and in dealing with patients with systemic diseases that may reduce the implant survival rate [49,50]. 

Our current study is limited in that all the included studies are in vitro. Therefore, further pre-clinical studies are warranted to establish more conclusive evidence regarding the antimicrobial effect of UV radiation on various implant surfaces.

## 5. Conclusions

In conclusion, UV irradiation on dental implant surfaces exhibited a significant antibacterial effect demonstrated through a reduction in bacterial attachment and biofilm formation with suppression of bacterial growth. Combination of UV and treated surfaces with alkali, plasma electrolytic oxidation, silver ion application, or heat enhance the overall photocatalytic antimicrobial effect. 

## Figures and Tables

**Table 1:** Detailed data of included studies. Risk of bias (11 low and 5 moderate).

Author/Year	Country	Effect	Material	Comparison	Assessment	UV Dose	Outcome
Naji S A/2018	Iran	Antimicrobial (*L. acidophilus*, *S. mutans* and *Candida albicans*)	Poly methyl methacrylate modified with hydrothermally synthesised titanium dioxide nanotubes.	UV-irradiated and non-irradiated marterial within the three groups of TiO_2_.	MICs, MBC, and MFC against planktonic microbial cells.	N/A	1/Significantly more antibacterial effects in the UV-irradiated disks than non-UV-irradiated disks (*p* value < 0.001).
Pantaroto H N/2018	Brazil	Antimicrobial (*S. sanguinis*, *A. naeslundii* and *F.nucleatum*)	Commercially pure titanium (cpTi) discs treated by radiofrequency mag-netron sputtering to obtain anatase (A-TiO_2_), rutile (R-TiO_2_) or mixture (anatase + rutile) (M-TiO_2_).	Different UV-A light exposure times (0, 1, 2, 3 and 4 h).	Biofilm assay and Biofilm Organization by scanning electronmicroscopy (SEM).	Treated UV-A light exposure (1 h) to generate reactive oxygen species production.	1/Significant antibacterial effect in A-TiO_2_ and M-TiO_2_ films on multispecies biofilm after 1 h of irradiation (*p* value < 0.001) with 99% and 99.9% reduction of bacterial counts, respectively.
Aung N/2019	Japan	Antimicrobial (*P. gingivalis*, *Prevotella intermedia*, *Fusobacterium nucleatum*, *A. actinomycetemcomitans*, and *S. oralis*)	Commercially pure titanium (cpTi) discs.	Different UV wavelengths	CFUs	UV light-emitting diodes with various wavelengths.	1/Powerful bactericidal effects (no bacterial colonies) with UV wavelengths of 265 and 285 nm.
Binns R/2020	USA	Antimicrobial (*Candida albicans*)	Poly (methylmethacrylate) resin.	UV and sodium perborate.	CFUs	UV light wavelength of 254 nm	1/Significant decrease in *C. albicans* survival with increasing ultraviolet light energy exposure (*p* = 0.00001). 2/Significant inhibition of *C. albicans* with UV of 254 nm treatment.
Cai Y/2013	Sweden	Antibacterial (*S. mutans*)	Noval nanopeptide (NP) adhesive.	UV-irradiated and non-irradiated and adhesive NP vs non-adhesive NP	Biofilms examination by SEM and metabolic activity assay.	UV light dose of 3 to 43 J/cm^2^.	1/Irradiation with 8.4 J/cm^2^ had a great reduction in the number of biofilm bacteria and a 5 times greater effect with 43 J/cm^2^. 2/UV-A dose of 16 J/cm^2^ is insufficient to affect the viability of biofilm.
Dini C/2020	Brazil	Antimicrobial (*S. sanguinis*)	Commercially pure titanium (cpTi) discs.	(1) Machined samples without UV, (2) PEO-treated samples without UV light application, (3) machined samples with UV light application, and (4) PEO-treated samples with UV light application.	CFUs	UV light wavelenght of 253.7 nm.	1/Significant decrease in the CFU counts for irradiated PEO than non-irradiated PEO (*p* = 0.012). 2/No significant difference in reducing the CFU counts between irradiated and non-irradiated cpTi (*p* = 0.269).
Han A/2018	China	Antimicrobial (*S. aures* and *P. gingivalis*)	Zirconia	Steam autoclave sterilization, dry heat sterilization, UV-C irradiation, and gamma (γ) ray irradiation.	CFUs	UV light with wavelength of 254 nm and 490 μW/cm^2^.	1/UVC and gamma ray irradiation increased the hydrophilicity of zirconia surface. 2/Dry-heat-sterilized samples showed the significantly lowest amount of bacteria growth than UVC and gamma ray irradiation.
Hatoko M/2019	Japan	Antimicrobial (*S. aures*)	Crystallized nanostructured titanium.	Formed by dark alkaline treatment heated at 600 C followed by UV-irradiated and non-irradiated Ti.	CFUs	UV light with wavelength of 254 nm, intensity of 100 mW/cm^2^.	1/UV irradiation decreased the viability of *S. aureus* up to 96% after 6 h. 2/No biofilm formation was obsereved on TNS-heat-UV after 18 and 24 h. 3/TNS-heat-UV inhibits bacterial attachment iferation, and biofilm formation.
Ishijima M/2019	USA	The oral microbial community culture.	Commercially pure titanium (cpTi) discs	UV-irradiated and non-irradiated	Biofilm formation examined by confocal lase scaning microscopy	UV light for 12 min	1/Significant low number of bacterial cells attached to irradiated surfaces than non-irradiated. 2/More biofilm thickness was noted with non-irradiation surfaces (16 μm) vs. irradiated less than 8 μm day 7). 3/Untreated titanium surfaces covered with significant more biofilm were 5-fold vs 2-fold rougher for irradiated surface.
Johnson H A/2020	USA	The attachment of *S. aureus*.	Different anodized commercially pure titanium grade 4 (CPTi4) surfaces.	Differing intensities UV irradiation	CFUs	UV irradiation (1 mW/cm^2^, 8 mW/cm^2^, and 23 mW/cm^2^)	1/Significant differences in bacterial attachment with reduction greater than 99% with irradiated by the 23 mW/cm^2^ UVA light.
Lee J E/2012	Korea	Antimicrobial (*S. sanguinis*) in the presence of saliva-coating.	Titunium machined (MA), heat-treated (HT), and anodized surfaces (AO).	MA vs. HT vs. AO, saliva-treated vs non-saliva-treated and UV-irradiated and non-irradiated materials.	CFUs	UV light of 2.0 mW/cm^2^ at a peak wavelength of 352 nm for 90 or 180 min.	1/UV-induced photocatalytic effects were significantly influenced by the presence of saliva-coating as well as by the crystal phase of the titanium. 2/Saliva-coating significantly increased the bacterial survival rates in the experimental and control groups.
SHIRAI R/2016	Japan	Antimicrobial (*P. gingivalis*)	Titanium dioxide (TiO_2_).	UV-irradiated and non-irradiated Ti.	CFUs	UV lights with wavelengths of 5 μm and 21 nm for 1, 3 and 6 h.	1/Significant reduction in number of *P. gingivalis* in both the 5 μm and 21 nm for 3 h vs. 0 h (*p* < 0.05). 2/Anatase TiO_2_ has an antimicrobial activity against periodontal pathogen.
Sun J/2020	China	Antimicrobial (*E. coli* or *S. aureus*)	Magnesium alloy with the single zinc oxide (ZnO) coating.	Different UV irradiation time for 0, 12 and 24 h.	CFUs	UV light of a 365 nm mercury lamp for 0, 12 and 24 h.	1/UV24h-ZnO had the highest inhibition of bacterial growth of cells (94.50 ± 1.25% against *S. aureus* and 98.95 ± 0.71% against *E. coli*) vs. UV0h-ZnO (82.47 ± 1.41% against *S. aureus* and 67.70 ± 1.32% against *E. coli*)
Tenkumo T/2020	Japan	Antimicrobial (The *S. mutans* or *A. actinomycetemcomitans*)	Commercially pure titanium (cpTi) discs.	Ag(+)L(+): Mixture of silver nitrate solution and bacterial suspension followed by UV-A light irradiation.	CFUs	UV-A light with wavelength of 365 nm and intensity of 1000 mW/cm^2^.	Significant higher bactericidal effect with combination treatment than silver ion application or UV-A light irradiation alone.
Yamada Y/2013	Japan	Antimicrobial (*S. aureus* or *S. pyogenes*)	Commercially pure grade 2 titanium discs.	UV-A or UV-C	Bacterial attachment or biofilm formation.	UV-A or UV-C intesity of 500 J/cm^2^.	1/Bacterial attachment, bacterial accumulation and biofilm formation were lower on irradiated surfaces than on the non-irradiated surfaces. 2/Irradiation with UV-C was superior to UV-A
Zhang H/2017	Japan	The attachment of Actinomyces ori.	Alkali-treated titanium with nanonetwork structures.	UV-irradiated and non-irradiated Ti.	CFUs	UV wavelength of 254 nm, intesity of 100 mW/cm^2^) for 15 min.	1/Reduced bacterial growth and inhibition of biofilm formation up to 6 h in irradiated TNS vs non-irradiated surfaces.
